# Multi-genome alignment for quality control and contamination screening of next-generation sequencing data

**DOI:** 10.3389/fgene.2014.00031

**Published:** 2014-02-20

**Authors:** James Hadfield, Matthew D. Eldridge

**Affiliations:** ^1^Genomics Core Facility, Cancer Research UK Cambridge institute, University of CambridgeCambridge, UK; ^2^Bioinformatics Core Facility, Cancer Research UK Cambridge institute, University of CambridgeCambridge, UK

**Keywords:** next-generation sequencing, quality control, contamination screen

## Abstract

The availability of massive amounts of DNA sequence data, from 1000s of genomes even in a single project has had a huge impact on our understanding of biology, but also creates several problems for biologists carrying out those experiments. Bioinformatic analysis of sequence data is perhaps the most obvious challenge but upstream of this even basic quality control of sequence run performance is challenging for many users given the volume of data. Users need to be able to assess run quality efficiently so that only high-quality data are passed through to computationally-, financially-, and time-intensive processes. There is a clear need to make human review of sequence data as efficient as possible. The multi-genome alignment tool presented here presents next-generation sequencing run data in visual and tabular formats simplifying assessment of run yield and quality, as well as presenting some sample-based quality metrics and screening for contamination from adapter sequences and species other than the one being sequenced.

## INTRODUCTION

It is vital in any laboratory to assess the quality of the data being generated. In a next-generation sequencing (NGS) facility the volumes of data can be overwhelming and automated quality control (QC) reporting is an ideal. There are many metrics to consider when looking at a sequencing run, some are run specific, others sample specific and many can be affected by both run and sample. Understanding what individual metrics mean in a particular context is complex and can require significant experience. Tools that help simplify analysis by building on this experience and removing subjectivity are becoming increasingly vital. We have developed the multi-genome alignment (MGA) contamination screen that can be used to calculate a few key, simple but important metrics, primarily data yield and quality, whilst also providing some additional sample related QC.

Tools like MGA are not new. Perhaps the earliest example of a QC tool is the Phred (Ewing et al., 1998) package developed to improve methods for gel-based Sanger-sequencing trace analysis and base quality scoring. The abstract of this paper written 15 years ago is surprisingly relevant today, stating: “it is particularly important that human involvement in sequence data processing be significantly reduced or eliminated” and that there is a need to “make human review (of sequence data) more efficient.” Almost every molecular biologist working today has seen and benefitted from this work in their Sanger sequencing results. Life Technologies (formerly Applied Biosystems, Santa Clara, CA, USA) produced a free tool, Sequence Scanner v1.0 (Applied Biosystems, 2005), that allowed a very quick visual check of 96 samples. This move away from inspection of individual traces to a more gross assessment of a Sanger sequencing run was necessitated by the increase in sample volumes due to the introduction of automated capillary sequencers. The introduction of microarrays was accompanied by QC tools that allowed the vast amounts of data to be assessed before starting complex analysis pipelines. Two of the major providers included such tools; Affymentrix (Santa Clara, CA, USA) provided a simple text-based reporting tool in their MAS5.0 (Hubbell et al., 2002) primary data analysis package and Agilent Technologies (San Francisco, CA USA) provided a very comprehensive visual, graphical, and text-based tool in their Feature Extraction software (Agilent, 2013). For NGS data the most widely used software, not provided by instrument vendors, for quality analysis of data is FastQC (Andrews, 2010), which presents multiple metrics for each dataset, including per base sequence quality score, per base GC content and duplication rate. A feature of the most of the tools above is their reliance on multiple metrics to report on the quality of what are complex assays. However, the use of individual metrics must be evaluated carefully alongside others including the starting sample QCs, and in the context of the sample or run being considered.

The MGA tool presented here aims to provide a subset of metrics that can be quickly assessed with minimal explanation, and allow users of NGS data to determine if the data generated are of sufficient yield and quality. The tool is not intended to be comprehensive nor used in isolation, rather as part of a formal assessment of experimental quality.

### MATERIALS AND METHODS

The MGA contaminant screen is an alignment-based method for detecting contamination in genomic or transcriptomic sequencing libraries. A sample of read sequences and base quality scores is extracted from the FASTQ files produced by the sequencing instrument. In practice, we have found that a sample of 100,000 reads is sufficient to detect moderately low levels of contamination. This represents a small fraction of the data usually generated from a lane of sequencing. The screen can be run on any number of FASTQ datasets so it would be feasible to look separately at every library in a multiplexed pool to determine the likely contamination in each. We generally assess contamination at the lane level on the basis that libraries from difference sources are not typically grouped together on the same lane.

Two differing alignment approaches are taken for (1) identifying sequences likely to have originated from a different species to that being sequenced and (2) detecting the presence of adapter sequences ligated to the ends of sequence fragments. The screen is not capable of detecting contaminant sequences from the same species as that being sequenced.

For detecting cross-species contamination the sampled reads are trimmed to 36 bases and aligned using bowtie (Langmead et al., 2009) to a set of reference genome sequences representing possible contaminants. This includes several mammalian species that are used in our laboratory as well as several thousand bacteria, viruses, and other microorganisms. The latter are grouped together so that, for example, the sampled reads are aligned to a collection of bacterial reference genome sequences and results reported for the set; consequently, the screen may detect bacterial contamination but will not be specific about the contaminant species. We choose to trim the read sequences so that the results from different sequencing runs can be compared and to derive baseline alignment and error (or mismatch) rates; trimming also helps reduce the computational cost and allows for detection of contaminants in runs with adapter contamination (see below). The reads are also aligned to the reference sequence for phi X 174 bacteriophage, commonly used as a spike-in control. Controls are differentiated from target species and contaminants in the final report.

The alignment results for each species, or collections of reference genomes in the case of bacteria, viruses, and fungi, are collated and species are ranked based on the number of reads aligning to each. Each read may align to multiple species as a result of sequence homology between species. To distinguish likely contamination from sequence homology each read is assigned to a single species based on the above ranking. For example, if the target species is human, some of the reads may also align to the mouse genome. Assuming that more reads align to the human reference sequence than that for mouse, all reads that align to both will be assigned to human, and only those that uniquely map to the mouse genome (and not another higher-ranked species) will be assigned to mouse.

The method for detecting sequencing adapters differs because it is possible that only a part of a read sequence is adapter. This can occur when the genomic fragment is shorter than the number of bases sequenced such that the sequencing runs through to adapter on the 3′ end. Accordingly, we report adapter contamination separately since sequences can be associated both with cross-species and adapter contamination. The sampled reads are first converted to FASTA format and then aligned to a set of adapter and primer sequences using the exonerate sequence alignment tool (Slater and Birney, 2005); this is run using a local alignment model with affine gaps, similar to the Smith–Waterman–Gotoh algorithm (Smith and Waterman, 1981; Gotoh, 1982).

**FIGURE 1:**
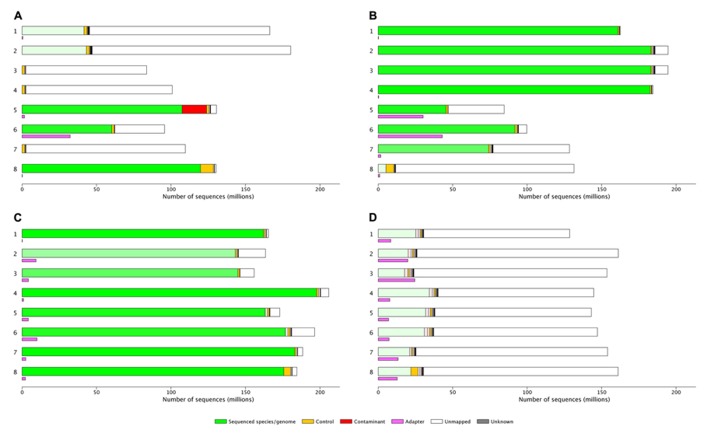
**(A)** 997M reads, lots of bad libraries and higher than expected PhiX indicating poor quantification of sequencing libraries: Lanes 1 and 2 user A: 25%A, 73%U, 3.5%E, > 1%Ad. Lanes 3,4 and 7 user B: 97%U, these libraries were from a species that was not included in the set of reference genomes aligned to using MGA, 1–2%P. Lanes 5,6 and 8 individual users, lane 5 shows 13% mouse genome contamination, lane 6 35%U, 34%Ad, lane 8 92%A, 0.2%E, 6.6%P. **(B) **1180M reads, quality can be attributed to different library preparations. Lanes 1–4 user A: 99%A, low error rate, and no adapter. Lane 5 user B: 53%A, 46%U, 37%Ad (a known problem with this library type). Lane 6 user C: 92%A, 5.8%U, 43%Ad. Lane 7 user D: 59%A, 0.55%E, 38%U. Lane 8 user D 91%U. **(C) **1432M read. Lane 1 user A: 98%A, 0.3%E. Lane 2 and 3 user B 87–93%A, 6–11%U, 0.6–0.8%E, 3–6%Ad. Lane 4 user C: 96%A, 0.3%E. Lane 5–8 user D: 90–97%A, 2–8%U, 0.2–0.36%E, 1–5%Ad. **(D) **1193M reads, a good flow cell with what look like poor libraries. Lanes 1–8 user A: 11–24%A, 72–84%U, 2.5–3.6%E, 5–16%Ad. %A, percent aligned to the target genome; %U, percent unmapped; %P, percent PhiX control genome; %E, error rate; %Ad, percent aligned to adapter sequences.

Results are presented in both a tabular and graphical form (**Figure 1** and supplementary file), the latter as a stacked bar chart in which each portion of the bar represents the assigned reads for a particular species. The bars are colored green if they match the target species, orange if they match the control, and red if they match another species. The transparency of the bars is adjusted depending on the error or mismatch rate of alignments for the species, with lower mismatch rates corresponding to more opaque bars drawing attention to likely contamination. Adapter contamination is displayed as a separate mauve bar.

The various sampling, trimming, conversion, alignment, and collation steps are defined in an analysis workflow and executed using an in-house workflow management system on a high-performance compute cluster. The MGA screen can also be run on a multi-processor server or high-end workstation. For a single dataset or lane, we align to reference sequences for 23 species and collections of several 1000 bacteria, viruses, and fungi. Each alignment job takes approximately 5 min and the results for eight lanes of an Illumina HiSeq-2000 flow cell are usually available within 15 min of the FASTQ sequence data being available on the compute cluster (overall CPU is around 3–4 hours).

The software, as well as instructions for installing and running MGA, are available here: https://github.com/crukci-bioinformatics/MGA

## RESULTS

The MGA tool has been used for every sequencing run performed at the Cambridge Institute genomics core for the past three and a half years, on Illumina’s (San Diego, CA, USA) GAIIx, HiSeq, and MiSeq platforms.

This graphical representation allows very quick estimation of the yield and quality of each flow cell or lane.

It is relatively simple to determine the difference between “good” and “bad” flow cells (**Figures 1C,D**), “good” and “bad” samples (**Figure 1B** lanes 1–4 vs lanes 5 and 6 or 7 or 8) or flow cells which will require significant investigation by the sequencing lab (**Figure 1A**) or the user (**Figure 1D**). However, the interpretation of results needs to be taken in context of the type of library or run, as either can significantly influence QC metrics. The flow cell shown in **Figure 1D** is known to be a reduced representation bisulfite sequencing (Meissner et al., 2005) run and the alignment of this data is expected to be poor; the high yield of this flow cell suggests the user will be happy with the results generated and no further investigation is likely to be necessary. Flow cell B (**Figure 1B**) lanes 5 and 6 show high adapter contamination of the sequencing lane; this is likely to point to issues with sample preparation in the laboratory where the samples originated. These examples demonstrate how MGA can facilitate sequencing users identification of issues with particular sequencing lanes/flow cells.

## DISCUSSION

The MGA contaminant-screen tool was originally conceived to answer queries about contamination in the sequencing process. Contamination can occur at any point along the sequencing process, in a research laboratory where samples are being extracted and libraries prepared, or in a sequencing facility where many thousands of libraries are being handled. An early analysis script simply interrogated the level of PhiX in each lane as we hypothesized that if contamination arose in the sequencing laboratory then PhiX, which should only appear in lane 8 (the control lane) would also be present in lanes 1–7 at variable levels. Analysis confirmed that significant PhiX contamination in lanes 1–7 was limited to a handful of flow cells.

The utility of the tool in this instance demonstrated how useful a similar approach would be as part of our routine QC of each sequencing lane. The use of a control lane increases sequencing costs by 12.5% and is no longer routine. We moved to a process of unbalanced loading of PhiX: 1% in lanes 1–7 and 5% in lane 8. This simple method allows us to detect any inversion of the flow cell, and to determine if low yield is the result of a sequencing or sample issue. If low yield is due to poor clustering/sequencing then the percentage of PhiX will be as expected, whereas if it is due to poor library quantification then the percentage of PhiX will be incorrect, in any low-yield lanes. This has become an important tool in deciding how and when to repeat sequencing runs with low yield, and determining who should pay for the repeat lane(s).

When designing the MGA visualization we considered the metrics most useful to determine the yield and quality of a particular sequencing run. As an Illumina run can contain one or two flow cells, and as most flow cell lanes contain a single sample, we present results in a per lane format. We also tried to consider the context that these reports might be used in and the limitations our methods might have. Illumina provide many QC metrics in their instrument control software. Commonly analyzed metrics are yield, percentage passed filters (%PF), error rate, phasing and prephasing, cluster density, and per cycle reporting of Q-score and percent Q30 data. The very commonly used FastQC tool provides a modular set of analyses that imports data from BAM, SAM, or FASTQ files and generates eleven summary plots including basic run statistics including number of reads, per base sequence quality, and duplicate sequences. A more recent tool is Illumina’s QC app (Illumina Inc, 2013), which generates an automated Library QC report containing several QC metrics in tabular and visual form. The MiSeq QC app incorporates a diversity estimate (Daley and Smith, 2013) for each sample that can be used to determine the limit of sequencing depth. The MGA primarily visualizes two details important to all users and managers of NGS data; yield and quality, it also presents data that can be useful in determining why a particular run/lane is sub-optimal in the accompanying tables.

Multi-genome alignment is one tool that core facility managers, bioinformaticians or users can use to assess their sequence data. The use of multiple tools can be confusing so in most cases users will limit themselves to one or two methods. However, there is not currently a single QC tool for NGS data that provides all the metrics users might require, and different types of user will require different tools at different times. MGA allows very quick interpretation of per lane yield and quality with minimal explanation, allowing the Genomics Core facility at the Cancer Research UK Cambridge Institute to inspect each of approximately 2000 lanes per year.

## Conflict of Interest Statement

The authors declare that the research was conducted in the absence of any commercial or financial relationships that could be construed as a potential conflict of interest.
